# More than a whistle: examining the role of organizational culture and talent development in football referee progression

**DOI:** 10.3389/fpsyg.2025.1701987

**Published:** 2025-12-05

**Authors:** Anthony Taylor, Mark Batey, Danny Powell, Abhijeet Patra, Andrew Denovan, Neil Dagnall

**Affiliations:** 1 Professional Game Match Official (PGMOL), Wembley, United Kingdom; 2 School of Psychology, Manchester Metropolitan University, Manchester, United Kingdom; 3 School of Psychology, Liverpool John Moores University, Liverpool, United Kingdom

**Keywords:** talent development, officiating bottleneck, referee retention, mental toughness, conscientiousness, organizational culture

## Abstract

Talent development is a foundational process that enables performance, facilitates the realization of potential, and promotes positive engagement with growth opportunities. In the context of football, this is important to referee bodies because of the need to attract, retain, and progress officials. Noting high referee attrition and a shortage of elite officials, this study examined how conscientiousness, mental toughness, and organizational culture influenced referees’ perceptions of talent development. One hundred and eighty-one referees, categorized by the highest level at which they had officiated, completed self-report measures. Analysis revealed a significant main effect of referee level on perceptions of talent development. Officials at intermediate level 4 reported a specific drop in talent development, particularly in Holistic Quality Preparation (integrated development) and Support Network (availability of assistance), indicating a developmental bottleneck. Further comparisons among the most experienced officials found that while conscientiousness and mental toughness did not differ, Super-Elite (i.e., international medal winning) versus Elite (i.e., high ranking) referees held significantly more positive perceptions of organizational culture and talent development. Thus, while a baseline level of psychological resources is essential for officiating, perceived quality of organizational environment distinguishes between those at the highest levels of refereeing.

## Introduction

At all levels, football match officials operate in challenging environments. Illustratively, Premier League referees evaluate approximately 245 discrete events per match, making on average a decision every 22 seconds (Brand, 2018). Most of these judgments directly influence match outcomes, such as assessing the fairness of physical contact or determining disciplinary sanctions. While such figures highlight the demands of officiating, referees overtly signal only around 35 decisions per match, leaving much of the decision-making process obscured. Hence, the disparity between high-frequency, implicit rulings and low-incidence explicit decisions results in intense external scrutiny and performance evaluation.

At the highest levels, Video Assistant Referee (VAR) protocols further intensify examination of on-field officials. Introduced into the Laws of the Game in the 2018/19 season (and debuting in the Premier League in 2019/20), VAR was designed to correct clear and obvious errors in match-changing situations. However, its implementation has added new layers of complexity, including in-game delays and novel communication demands, and has heightened focus on specific incidents (Sánchez Cid and García, 2020; Pina et al., 2021). Despite these technological advances, and underscoring the demands of officiating, error rates among elite refereeing cohorts remain around six per match (Van Biemen et al., 2022).

Beyond cognitive and technological pressures, football officiating requires a high standard of physical fitness. Referees at high levels often exceed 11 km of high intensity running per match (Bloß et al., 2020; Helsen and Bultynck, 2004). Additionally, referees at all levels routinely experience negative interactions with players, coaches, spectators, and receive criticism via social media. This is especially problematic since they typically receive only limited preparation and support for handling challenging encounters (Potrac et al., 2021). These multifaceted and evolving exigencies highlight why continuous development and adaptation are essential for football officials.

Given the inherent challenges of refereeing, the ability to withstand pressure and effectively utilize training and support mechanisms is crucial for referee recruitment, retention, and progression (Cunningham et al., 2022). Although referees are indispensable to the organization of competitive fixtures, with matches unable to take place without them (Webb, 2022), national associations continue to face an acute shortage of top officials (Ornstein, 2022). Research has attributed this shortage to limited administrative support and the prevalence of verbal abuse from players, coaches, and spectators (Giel and Breuer, 2020; Mojtahedi et al., 2024; Webb et al., 2020).

Consequently, many referees experience declining motivation and inadequate developmental support, leading to high attrition rates, particularly during the formative stages of their careers (Ali et al., 2023; Forbes and Livingston, 2013). While initiatives such as mentoring programs and full-time contracts, introduced by the Professional Game Match Officials Ltd. (PGMOL) in England, represent important steps forward, turnover and progression bottlenecks persist, highlighting the need for more comprehensive and sustainable talent development structures across all levels of football officiating (see Slack et al., 2013, 2015).

Accordingly, research exploring referee perception of development opportunities is critical (Cunningham et al., 2024; Webb et al., 2025; Webb et al., 2023). However, investigations in this domain remain limited due to practical barriers (i.e., demanding training and playing schedules) and the privacy concerns of higher-level professional referees. This research gap is significant because referee decisions directly influence match outcomes, have financial implications for clubs, and impact on perceived sporting integrity (Cunningham et al., 2024; Westbrooks et al., 2023).

Recognizing environmental demands, shortage of officials, and conceptual gaps, this study explored the impact of conscientiousness and mental toughness on referees’ perceptions of talent development opportunities. The authors selected these psychological constructs because they are strongly associated with performance across demanding, real-world settings, including academic, organizational, and sports contexts (see conscientiousness: Minnigh et al., 2024; Potočnik et al., 2021; Shuai et al., 2023; mental toughness: Cross et al., 2024; Dagnall et al., 2021; Denovan et al., 2023a; Denovan et al., 2023b).

Conscientiousness reflects goal-focus, dependability, and persistence toward achieving goals. In sport, conscientiousness predicts coach ratings of football performance (e.g., athletic ability and work ethic) and objective game indices (e.g., goals, assists) (Piedmont et al., 1999). This signifies that conscientious individuals, as characterized by diligence, a sense of duty, and discipline, are more likely to excel and maintain exacting standards, which are principal features of effective officiating (Lampe et al., 2024; O’Brien et al., 2023). Further supporting this, research on expert handball referees found elevated levels of conscientiousness linked to meticulous pre-match preparation and rule fidelity (Dodt et al., 2022).

Researchers consistently link mental toughness, defined as an individual’s capacity to remain committed, focused, and resilient when facing challenges, pressures, and adversity (Dagnall et al., 2021; Denovan et al., 2023a), to high-level sporting accomplishment (e.g., Anthony et al., 2016; Jones et al., 2002, 2007). Evidence from football shows players at higher competitive levels report greater mental toughness (Grønset et al., 2024; Kristjánsdóttir et al., 2019; Pettersen et al., 2022).

Among officials, Slack et al. (2013) found that Premier League referees with higher levels of mental toughness coped more effectively with match-day pressures, media scrutiny, and post-decision challenges, and were better able to manage conflict with players and coaches. This aligns with broader evidence that mental toughness supports individuals in navigating the inherent pressures of high-level sport, making it a crucial attribute for both elite athletes and officials (Wheatley et al., 2023; Cunningham et al., 2025; Mojtahedi et al., 2023; Robazza et al., 2025).

Although mental toughness is a vital psychological resource, due to theoretical overlap with other non-cognitive constructs (e.g., grit, ego resiliency, and self-efficacy; Denovan et al., 2023b; Yankov et al., 2019) and contextual variations, it is difficult for theorists to determine its unique contribution to performance variance. Moreover, critics caution against the uncritical adoption of mental toughness as a positive trait because this interpretation masks potential contributions to maladaptive coping strategies and obscures the organizational and environmental factors that impact upon performance (see Gucciardi, 2017; Lin et al., 2017; Papageorgiou et al., 2019). Nonetheless, from a well-being perspective, mentally tough individuals report lower levels of depression, anxiety and stress (Drinkwater et al., 2019; Mojtahedi et al., 2021).

To address these conceptual concerns, this study adopted a multi-construct approach examining mental toughness alongside the foundational personality trait of conscientiousness. Examining these constructs in tandem was advantageous because conscientiousness, as a stable and well-validated trait, anchors and clarifies variance shared with mental toughness, such as persistence and goal-directedness. This allows theorists to isolate the unique contributions of mental toughness by controlling for variance accounted for by conscientiousness (i.e., self-discipline, perseverance, and goal-striving) (Roberts et al., 2014). Additionally, as both mental toughness and conscientiousness correlate positively with agreeableness and negatively with the Dark Triad (Liang et al., 2024), construct concurrency highlights their shared role as adaptive psychological resources (Taberner and MacQuarrie, 2025).

This interpretation aligned with the “4 C’s” model of mental toughness (Clough et al., 2002): Conscientiousness underpins *commitment* through goal-striving, *control* via fostering self-discipline, *challenge* by promoting obstacle negotiation, and supports *confidence* through consistent application. Thus, conscientious individuals are more likely to develop and utilize psychological resources central to mental toughness, such as resilience and motivation.

Research on Big Five traits confirms a moderate to strong positive correlation between conscientiousness and mental toughness (Delaney et al., 2015; Yankov et al., 2019). Furthermore, research with football players by Rodrigues et al. (2023) established that conscientiousness, via mental toughness, indirectly predicted performance. This designates that while conscientiousness drives initial effort and goal-focus, it is mental toughness, fostered by conscientiousness, which enables individuals to translate effort into sustained performance, making these traits pertinent to referee development (from both attitudinal and engagement perspectives).

Thus, examining conscientiousness and mental toughness concurrently was advantageous, as these constructs capture the interplay between enduring personality traits and performance-related psychological resources that contribute to referee development. Understanding how they influence perceptions of talent development opportunities offers insight into the individual and organizational factors that support retention, progression, and sustained performance under pressure.

Beyond individual psychological traits, referee perceptions of their officiating body’s organizational culture profoundly shape their development and progression (Ali et al., 2023; Webb et al., 2023). Organizational culture refers to the shared values, beliefs, attitudes, norms, and practices that influence training, learning, assessment, and interaction. A positive organizational culture fosters psychological safety, trust, and coherence in feedback and support systems, which in turn enhance motivation and resilience, facilitating development (Hauser et al., 2024; Martins and Terblanche, 2003). For example, Webb et al. (2023) found regional variations in mentoring quality and perceived support networks directly impacted referee promotion prospects and stress levels.

Perceived organizational support, which denotes the extent to which referees feel valued and supported by their organization (Ali et al., 2023; Kurtessis et al., 2017), is closely related to organizational culture. When officials perceive strong mentorship and growth opportunities, they tend to reciprocate with loyalty, effort, and sustained engagement (Caesens and Stinglhamber, 2020; Shanock et al., 2019). Conversely, poorer perceptions of support, particularly among senior referees, link to dissatisfaction, disengagement, and discontinuation (Choi and Chiu, 2017; Forbes and Livingston, 2013).

Talent development, in the context of performance, is the ability to acquire expertise without regard to the process (Collins and MacNamara, 2017). While sports research traditionally focused on physiological and perceptual-cognitive factors in talent identification and development (TID; Baker et al., 2020), contemporary approaches view the process as dynamic, socio-cultural, and non-linear. These perspectives highlight the interplay between deliberate practice, psychosocial support, organizational culture, and individual dispositional characteristics (Güllich and Larkin, 2023; Höner et al., 2023). Despite theoretical advances, the developmental trajectories of officials remain under-researched. Traditional football TID systems, often reliant on short-term performance indicators, possess only modest predictive validity beyond one or two seasons (Baker et al., 2017; Vaeyens et al., 2008), suggesting they are insufficient for long-term talent identification. The unique dual-career timelines referees face, commonly balancing employment and officiating until their mid-thirties (Cunningham et al., 2024), further complicate progression structures by compressing the deliberate practice opportunities typically afforded to full-time athletes.

Qualitative research confirms that English football referees often perceive development pathways as lacking transparency and consistent organizational support, including limited mentorship opportunities (Webb et al., 2023). Beyond systemic issues, existing talent development approaches overemphasize knowledge and skills relevant only to performance contexts (Kittel et al., 2019; Mojtahedi et al., 2024; Russell et al., 2025), leaving crucial constructs like non-cognitive skills and perceptions of organizational culture empirically underrepresented. This omission limits the capacity for evidence-based recruitment and development strategies in officiating (Baker et al., 2020), underscoring the need for research that integrates psychological characteristics and organizational context to better understand pathways to elite refereeing.

## The present study

The authors hypothesized that perceptions of Athletic Talent Development (ATD) would change as referees progressed through levels. For example, the transition from local football matches (Level 5) to regional semi-professional leagues (Level 4), where individuals can also act as assistant referees for Level 3 games (National League System), presents distinct challenges likely to affect developmental focus. Research shows that transition points, such as those between competitive levels, are affiliated with a lack of support and increased dropout rates (Giel and Breuer, 2020; Mojtahedi et al., 2024; Webb et al., 2020). At advanced levels, characteristics like aligned expectations and clear communication should become increasingly salient. Acknowledging the limited prior research in this domain, our study sought to identify differences for subsequent research to examine.

Prior research reports that conscientiousness and mental toughness are positively related to ATD perceptions (Cowden, 2017; Li et al., 2019; Rodrigues et al., 2023; Shuai et al., 2023; Tedesqui and Young, 2020; Weinberg et al., 2016). We argue these traits are not just performance indicators but fundamental enablers of developmental engagement. Conscientiousness, through diligence and a drive for perfection, links to consistent effort and continuous learning, leading to a more positive perception of developmental support as individuals actively utilize resources. Simultaneously, mental toughness, by providing psychological resources to endure pressures, enables effective engagement with talent development opportunities. Without it, even conscientious referees may struggle, leading to disengagement and less favorable perceptions of self-advancement and organizational support. Additionally, we expected referee views of organizational culture would influence ATD perceptions (e.g., Ali et al., 2023; Webb et al., 2023), with a positive and supportive climate fostering positive perceptions of development opportunities.

Beyond individual contributions, it is plausible that conscientiousness, mental toughness, and organizational culture inter-relate and jointly influence perceptions of talent development. For instance, a highly conscientious, mentally tough referee who views the organizational culture positively may be better able to capitalize on talent development opportunities, regardless of level.

While a primary aim was to examine how ATD perceptions differ across referee levels, this study also recognized the importance of individual psychological attributes and the broader organizational environment. Therefore, beyond investigating their direct relationships with development perceptions, we controlled for their potential confounding effects. This approach allowed us to explore these relationships while isolating the unique contribution of referee level after accounting for individual and contextual influences.

A further consideration of this paper was to determine whether referees at the highest tiers of officiating (i.e., National Elite and FIFA; Elite and Super Elite) differed in terms of psychological attributes (i.e., conscientiousness and mental toughness) and perceptions of the developmental environment (i.e., organizational culture and ATD). Given that conscientiousness and mental toughness are fundamental attributes of effective refereeing, we predicted that officials at all levels would demonstrate similarly high levels. However, given that the quality and intensity of organizational support and developmental structures typically increase with progression, we hypothesized that referees at the highest (vs. high) levels would rate organizational culture and ATD more positively.

**Figure 1:**
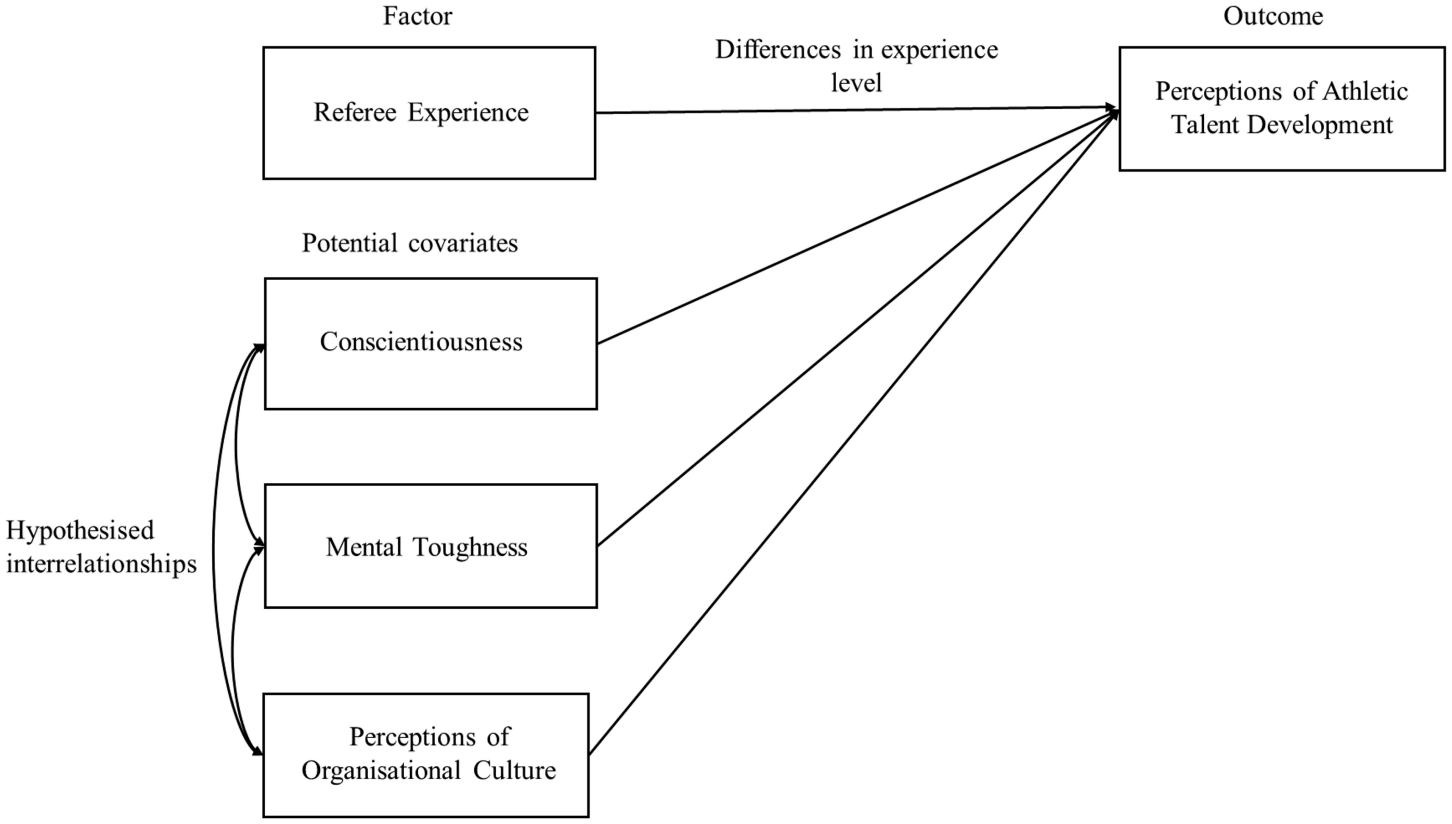
Conceptual model depicting the hypothesized relationships and interactions with regards to referee level, conscientiousness, mental toughness, organizational culture, and athletic talent development.

The theoretical framework driving this study posits that while individual psychological attributes (conscientiousness and mental toughness) are fundamental enablers of engagement with refereeing, organizational support (culture) and the challenges of progression stages (referee level) jointly influence perceptions of ATD. Based on this framework, we tested the following hypotheses: Perceptions of ATD would differ across refereeing levels (H1); with conscientiousness, mental toughness, and organizational culture correlating positively and acting as significant covariates for ATD (H2); conscientiousness and mental toughness would not differ between the most accomplished officials (FIFA Super-Elite vs. Elite; FIFA and National) (H3); and FIFA Super-Elite (vs. Elite; FIFA and National) referees would report more positive perceptions of organizational culture and ATD (H4). Figure 1 diagrammatically presents the model underpinning this research.

## Method

### Participants

One hundred and eighty-one participants (175 male and 6 female) took part in this study. To ensure participant anonymity, the researchers collected only age group information. Participant ages were categorized according to the recommendations of Webb et al. (2025), and were as follows: 18–24, *n* = 36 (19.90%), 25–34, *n* = 67 (37.00%), 35–44, *n* = 37 (20.40%), 45–54, *n* = 21 (11.60%), 55–64, *n* = 14 (7.70%), and 65 plus, *n* = 6 (3.30%).

#### General classification

Participants either currently officiated or had previously officiated. The researchers leveraged the established networks of one of the authors. This produced a combination of convenience and snowball sampling, which enabled the researchers to access both a range of officials and a cohort of hard-to-access elite referees.

In terms of performance level the sample comprised: Trainee to Level 5 (trainee is undergoing initial refereeing education and Level 5 can officiate local county football matches), *n* = 41 (22.70%); Level 4 (can officiate regional semi-professional leagues, and act as assistant referees for Level 3 games), *n* = 92 (50.80%); Level 3 (can officiate in the National Contributory League System and act as assistant referees for Level 2 matches), *n* = 16 (8.80%); Level 2 (can officiate in the National League North and South), *n* = 8 (4.40%); National List Elite (can officiate professional game matches in the Premier and Football League), *n* = 12 (6.60%); and FIFA (selected by their national association to officiate international FIFA and UEFA matches), *n* = 12 (6.60%). Regarding years actively qualified, interquartile range was 5.00 to 13.00 years; mean duration 11.00 (SD = 8.87) years.

#### Elite classifications

For analysis, the researchers applied a refined classification to the most accomplished officials. This derived from the conceptual framework of Güllich et al. (2019), who defined super-elite athletes as international medal winners. Applying this, distinguished between Elite (National, *n* = 12; FIFA, *n* = 7) and Super-Elite (FIFA, *n* = 5) referees.

Regarding FIFA, FIFA Elite have officiated in international matches organized by FIFA and UEFA, such as Champions League, Europa League, and National team matches. FIFA Super-Elite have officiated major international tournaments and finals such as the World Cup, EURO, and club competition finals. This detailed, two-tiered classification provided a precise, conceptually sound basis for examining experience differences in high-level officials. See Table 1 for a summary of participant characteristics.

**Table 1:** Summary of participant characteristics (*N* = 181).

Characteristic	Category	*n*	%
Total sample size	All participants	181	100.0
Gender	Male	175	96.7
	Female	6	3.3
Age group (years)	18–24	36	19.9
	25–34	67	37.0
	35–44	37	20.4
	45–54	21	11.6
	55–64	14	7.7
	65+	6	3.3
Referee level (progression)	Trainee to Level 5	41	22.7
	Level 4	92	50.8
	Level 3	16	8.8
	Level 2	8	4.4
	National Elite	12	6.6
	FIFA International	12	6.6
Referee level (analysis groups)	Low (Trainee to L5)	41	22.7
	Intermediate (Level 4)	92	50.8
	Higher (Level 3+)	48	26.5
Expertise (high-level analysis)	National Elite (NE)	12	50.0
	FIFA Elite (FE)	7	29.2
	FIFA Super Elite (FSE)	5	20.8

Percentages are valid responses. “Referee Level (Progression)” represents official grading levels within the referee development pathway. “Referee Level (Analysis Groups)” and “Expertise (High-Level Analysis)” denote aggregated groupings used for subsequent analyses.

### Measures

The researchers assessed mental toughness, conscientiousness, organizational culture, and athletic talent development using self-report measures.

### Mental toughness

The 10-item Mental Toughness Questionnaire (MTQ10) was employed in this study as an abridged, unidimensional measure of mental toughness (Dagnall et al., 2019; Papageorgiou et al., 2018). Practical considerations such as participant time constraints and limited access to referees for data collection informed selection of the MTQ10, over the longer 48-item MTQ-48 (Clough et al., 2002).

Furthermore, due to psychometric superiority (Dagnall et al., 2019) the researchers selected the MTQ10 over the earlier abridged 18-item version (MTQ-18; Clough et al., 2002). The MTQ-48 (and by extension, its abridged versions) assesses the capacity to handle pressure and recover from setbacks via four primary factors: Control, Commitment, Challenge, and Confidence. Participants respond to statements (e.g., “I generally feel that I am a worthwhile person”) using a five-point Likert scale (1 = strongly disagree to 5 = strongly agree). Total scores range from 10 to 50, with higher scores indicating greater mental toughness. The MTQ10 has demonstrated satisfactory reliability across various methods, including composite, alpha, and test–retest (Dagnall et al., 2019; Denovan et al., 2021, 2024; Papageorgiou et al., 2018).

### Conscientiousness

The Concise Conscientiousness Measure-Short Version (CCM-S; Franzen et al., 2022) is a 28-item instrument that assesses seven facets of conscientiousness: Industriousness, Control, Perfectionism, Task Planning, Tidiness, Procrastination Refrainment, and Caution. Within this study, due to their relevance to the competencies and challenges of sports officiating, the researchers measured only the Industriousness, Control, Perfectionism, and Task Planning dimensions. Investigators developed the CCM-S by adapting and shortening the Concise Conscientiousness Measure (MacCann et al., 2009). Correspondingly, the scale is valid and reliable at both global and facet levels (Franzen et al., 2022).

Industriousness reflects a referee’s work ethic, diligence, and commitment, encompassing preparedness, consistent effort, and continuous learning. Control pertains to self-regulation, composure, and the ability to make rational, impartial, and anticipatory decisions under pressure. Perfectionism, when adaptive, drives meticulous rule application, error detection, and striving for accurate, consistent decision-making. Task Planning is crucial for pre-match preparation, strategic positioning, anticipating game scenarios, and effective communication to manage game flow. Selection of these facets, since they corresponded with the core competencies of officiating, maximized construct contextual relevance (i.e., face validity).

The researchers omitted the remaining facets, Tidiness, Procrastination Refrainment, and Caution, as they are less applicable to the real-time demands of refereeing performance. Specifically, personal tidiness does not impact on-field decision-making, and the instantaneous nature of officiating leaves no room for task postponement (procrastination). Furthermore, an excessive tendency toward caution, as opposed to rapid, assured judgment, is detrimental to a dynamic game environment. Thus, for an investigation focusing on refereeing performance, prioritizing dimensions that contribute to rule enforcement, game management, and decision-making under pressure was appropriate.

Each facet comprises four items, assessed via a 5-point Likert-type scale (1 = not at all like me to 5 = very much like me), where responses indicate level of agreement with each statement (e.g., “I am always prepared”). Higher scores designate greater conscientiousness.

### Organizational culture

The Organizational Culture Survey (OCS; Glaser et al., 1987) assesses perceptions of organizational environment, including prevailing norms and values. Despite the availability of various organizational culture assessment instruments, the researchers selected the OCS due to its proven track record and established psychometric properties. The OCS provides clear and actionable dimensions: Teamwork, Information Flow, Morale, Employee Involvement, Supervision, Meetings, and Customer Service. These dimensions are pertinent for understanding the cultural dynamics within refereeing organizations. Furthermore, the adoption of the OCS enabled comparisons between findings and historical data.

The OCS comprises 31 items which appear as statements (e.g., “People I work with are direct and honest with each other”). Participants respond on a five-point scale Likert-type scale, ranging from 1 = strongly disagree to 5 = strongly agree. The present study assessed teamwork and conflict (6 items: i.e., interpersonal dynamics and collaboration), climate and morale (5 items, i.e., motivation, respect, fairness, trust, and efficiency), information flow (4 items, i.e., effectiveness and transparency of data flow), involvement (4 items, i.e., influence in organizational decision-making and the valuing of their ideas), supervision (7 items, i.e., supervisor’s effectiveness in communication, feedback, and delegation), and meetings (5 items, i.e., effectiveness and productivity of meetings within the organization). The OCS is valid and internally reliable (Erwee et al., 2001; Glaser et al., 1987).

### Athletic talent development

The Talent Development Environment Questionnaire (TDEQ; Martindale et al., 2010) assesses the ability to utilize talent development pathways within sport. Specifically, athlete perceptions and attitudes to aspects of the developmental environment. In this study, the TDEQ enabled the researchers to assess experienced quality of development, highlighting factors beyond on-field performance. This study used the 25-item, five-factor structure (TDEQ-5) identified by Li et al. (2015) because their model has attested measurement properties (i.e., convergent validity, discriminant validity, group invariance, reliability, and validity).

The TDEQ-5 evaluates five dimensions of athlete development, each measured by five items. These include Long-Term Development, focusing on sustained success through fundamental training, continuous opportunities, and a de-emphasis on immediate wins. Alignment of Expectations ensures coherent and individualized goal setting and review for sport development. Communication highlights effective dialog between coaches and athletes regarding development paths, training rationale, and feedback. Holistic Quality Preparation considers in-sport and out-of-sport development, emphasizing caring coaches, mental readiness, and a balanced life. Support Network provides comprehensive assistance from professionals, mentors, and coaches. Items within the TDEQ-5 appear as statements (e.g., “I am involved in decisions about my development”) and respondents indicate their level of agreement on a 5-point Likert-type scale (1 = strongly disagree to 5 = strongly agree). The TDEQ-5 has established psychometric properties (i.e., validity and reliability) (Brazo-Sayavera et al., 2017; Li et al., 2018; Thomas et al., 2020).

Within this study, measures demonstrated acceptable to excellent internal reliability, with Cronbach’s α and McDonald’s *ω* values ranging from 0.72 to 0.90. Specifically, reliability estimates were as follows: MTQ10 α = 0.73, ω = 0.73; CCM-S α = 0.86, ω = 0.86; OCS scale total α = 0.96, ω = 0.96; Teamwork and Conflict α = 0.87, ω = 0.87; Climate and Morale α = 0.89, ω = 0.89; Information Flow α = 0.79, ω = 0.80; Involvement α = 0.89, ω = 0.90; Supervision α = 0.90, ω = 0.90; Meetings α = 0.87, ω = 0.87; and TDEQ-5 Long-Term Development Focus α = 0.78, ω = 0.79; Alignment of Expectations α = 0.79, ω = 0.79; Communication α = 0.89, ω = 0.90; Holistic Quality Preparation α = 0.79, ω = 0.78; and Support Network α = 0.72, ω = 0.73.

### Procedure

After receiving institutional ethics approval, the researchers emailed eligible participants detailing the study’s purpose and nature. They obtained informed, voluntary consent by providing participants with a participant information sheet and a consent form, which participants signed and returned. Before data collection, the researchers pilot-tested the survey using a Think Aloud protocol. The lead researcher (a Super Elite Referee) and a test respondent verbalized their thoughts while completing the survey. This helped them evaluate the question difficulty and questionnaire length (Wolcott and Lobczowski, 2021), and they amended the order of some questions to improve the survey’s logical flow. They collected data using Qualtrics software. The researchers anonymised all data and kept it confidential on a password-protected computer.

The Manchester Metropolitan University Research Ethics Committee (ID: 57588) granted ethical approval. The researchers conducted the study in accordance with the university’s ethical guidelines.

### Analytical plan

Data screening and inspection of descriptive statistics and zero-order correlations occurred prior to performing multivariate analysis of covariance (MANCOVA). MANCOVA examined differences in Athletic Talent Development (ATD) as a function of level (i.e., stage of structured referee progression): Trainee to Level 5, Level 4, and Higher. Methodological and practical considerations informed the investigators decision to group Level 3 and higher officials into a single category. Specifically, merging acknowledged that because Level 3 demarcates progression to high-tier semi-professional and professional officiating, it is a critical transition point in referee progression, Furthermore, grouping addressed sample size limitations for individual high-level categories. Level 3 and above comprised small numbers, which restricted the ability to perform comparisons. Thus, the grouping ensured greater statistical power for the overall analysis, while permitting examination of differences as a function of referee level of progression.

Using MANCOVA enabled the researchers to investigate the effect of level on Athletic Talent Development (ATD), independent of Organizational Culture, Conscientiousness, and Mental Toughness. Given the known influence of these factors on ATD and the correlations observed in this study between the constructs and ATD dimensions (dependent variables), the researchers included Organizational Culture, Conscientiousness, and Mental Toughness as covariates (statistical controls). This approach controlled for their potential variance, increasing the precision and power of the main effect test. Following MANCOVA, to identify specific ATD dimensions driving the effects, the researchers further investigated significant main effects using Univariate ANCOVA. Finally, pairwise comparisons with Bonferroni correction (*p* < 0.05) pinpointed group differences while controlling for Type I error rates across multiple comparisons.

Following overall comparisons, analysis explored differences between Super-Elite and Elite officials. This involved a series of one-way independent ANOVAs (level: FIFA Super-Elite vs. FIFA Elite vs. National Elite). Planned comparisons then examined differences between: FIFA Super-Elite vs. FIFA Elite; FIFA Elite vs. National Elite; and FIFA Super-Elite vs. FIFA Elite and National Elite.

## Results

### Associations among key variables

Pearson correlations explored associations between Conscientiousness, Mental Toughness, Organizational Culture, and Athletic Talent Development (Long-Term Development Focus, Alignment of Expectations, Communication, and Support Network). Correlations, alongside descriptive statistics, appear in Table 2. For significant correlations, the researchers interpreted effect sizes using the recommendations from Gignac and Szodorai (2016), where a coefficient of 0.10 indicates a small effect, 0.30 a typical effect, and 0.50 a relatively large effect.

**Table 2:** Associations among study variables.

Variable	*M*	*SD*	1	2	3	4	5	6	7	8
1. CCS-M	4.03	0.46		0.26**	0.18*	0.28**	0.29**	0.35**	0.14*	0.26**
2. MTQ10	3.63	0.46			0.12*	0.08	0.13*	0.06	0.12	0.12
3. OCS	3.38	0.65				0.51**	0.45**	0.38**	0.34**	0.41**
4. LTDF	3.55	0.60					0.71**	0.69**	0.45**	0.65**
5. AoE	3.37	0.75						0.73**	0.54**	0.71**
6. Comm	3.38	0.81							0.39**	0.61**
7. HQP	3.35	0.76								0.63**
8. SN	3.48	0.68								

CCS-M, Concise Conscientiousness Measure; MTQ10, 10-item Mental Toughness Questionnaire; OCS, Organizational Culture Survey; LTDF, Long-Term Development Focus; AoE, Alignment of Expectations; Comm, Communication; HQP, Holistic Quality Preparation; SN, Support Network; *p < 0.05; **p < 0.001.

All significant correlations were positive. Specifically, Conscientiousness, Mental Toughness, and Organizational Culture inter-related. Conscientiousness and Organizational Culture associated with Athletic Talent Development dimensions, while Mental Toughness only related to Alignment of Expectations. Finally, Athletic Talent Development dimensions correlated.

Analysis included CCM-S and OCS as covariates within the MANCOVA to control for effects on Athletic Talent Development. Due to predominantly non-significant associations the authors omitted Mental Toughness. Homogeneity of covariance matrices (Box’s M *p* = 0.229) and variances existed (Levene’s *p*s > 0.076). These findings satisfied basic assumptions. MANCOVA indicated a significant main effect of referee experience, Pillai’s Trace = 0.14, *F*(10, 346) = 2.58, *p* = 0.005, η^2^ = 0.07 (medium effect). A significant effect of CCM-S (as a covariate) existed, Pillai’s Trace = 0.10, *F*(5, 172) = 3.62, *p* = 0.004, η^2^ = 0.09 (medium effect). A significant effect also occurred for OCS as covariate, Pillai’s Trace = 0.26, *F*(5, 172) = 12.47, *p* < 0.001, η^2^ = 0.26 (large effect). Univariate analyses (Table 3) revealed a significant effect in relation to Long-Term Development Focus, *F*(2, 176) = 5.32, *p* = 0.006, η^2^ = 0.06 (medium effect), Alignment of Expectations, *F*(2, 176) = 7.44, *p* < 0.001, η^2^ = 0.08 (medium effect), Communication, *F*(2, 176) = 3.09, *p* = 0.048, η^2^ = 0.03 (small effect), Holistic Quality Preparation, *F*(2, 176) = 3.47, *p* = 0.033, η^2^ = 0.04 (small effect), and Support Network, *F*(2, 176) = 9.49, *p* < 0.001, η^2^ = 0.10 (medium effect).

**Table 3:** ANCOVA and pairwise comparisons for athletic talent development among football referees.

Variable/comparison	LTDF, *F*(2, 176)	AoE, *F*(2, 176)	Comm, *F*(2, 176)	HQP, *F*(2, 176)	SN, *F*(2, 176)
ANCOVA					
Referee level	5.33*	7.45**	3.10*	3.47*	9.49**
CCS-M (covariate)	8.49*	9.30*	17.40**	1.04	6.96*
OCS (covariate)	56.21**	38.11**	24.36**	18.67**	30.47**
Pairwise comparisons					
Trainee to Level 5 (County) vs. Level 4 (Supply League)	0.75	1.31	0.56	1.71*	1.70*
Trainee to Level 5 (County) vs. Level 3 to FIFA International referee	1.44*	2.13**	1.02	0.76	0.36
Level 4 (Supply League) vs. Level 3 to FIFA International referee	0.69	0.82	1.58*	0.95	2.06**

CCS-M, Concise Conscientiousness Measure; OCS, Organizational Culture Survey; LTDF, Long-Term Development Focus; AoE, Alignment of Expectations; Comm, Communication; HQP, Holistic Quality Preparation; SN, Support Network; *p < 0.05; **p < 0.001.

Pairwise comparisons (post-hoc) revealed significantly lower Long-Term Development Focus and Holistic Quality Preparation scores for Level 4 referees than Level 5 to trainees (*p* = 0.005 and *p* = 0.036 respectively). Moreover, significantly higher Alignment of Expectations and Communication scores existed for Level 3 to FIFA International Level than Level 4 referees (*p* < 0.001 and *p* = 0.041 respectively). For Support Network, significantly higher scores existed for both Level 5 to trainees and Level 3 to FIFA International Level vs. Level 4 referees (*p* = 0.008 and *p* < 0.001 respectively) (Table 3).

### Group differences by referee expertise

One-way independent ANOVAs examined whether CCS-M, MTQ10, OCS, and ATD differed as a function of expertise (level: FIFA Super-Elite vs. FIFA Elite vs. National Elite). Planned comparisons examined differences between Super-Elite vs. FIFA (contrast 1), FIFA Elite vs. National Elite (contrast 2), and FIFA Super-Elite vs. Elite [FIFA and National (contrast 3)]. Descriptive statistics appear in Table 4.

**Table 4:** Conscientiousness, mental toughness, organizational culture, and athletic talent development scores relating to referee level.

Variable	Officiating classification							
	FSE (*n* = 5)		FE (*n* = 7)		NE (*n* = 12)		Total (*n* = 24)	
	*M*	*SD*	*M*	*SD*	*M*	*SD*	*M*	*SD*
CCS-M	65.20	8.70	63.00	5.32	64.08	8.54	64.00	7.49
MTQ10	39.80	2.77	39.43	3.51	36.83	4.80	38.21	4.19
OCS	127.00	19.27	98.71	18.61	90.25	23.05	100.38	24.85
ATD	102.80	11.32	82.29	15.52	82.75	15.68	86.79	16.53

FSE, FIFA Super Elite; FE, FIFA Elite; NE, National Elite; CCS-M, Concise Conscientiousness Measure; OCS, Organizational Culture Survey; ATD, Athletic Talent Development.

ANOVA found significant differences on OCS, *F*(2, 21) = 5.351, *p* = 0.013 and ATD *F*(2, 21) = 3.647, *p* = 0.044. Regarding OCS, FIFA Super-Elite (vs. FIFA Elite), *t* = 2.282, *p* = 0.017, *d* = 1.64; and (vs. FIFA and National Elite), *t* = 3.033, *p* = 0.003, *d* = 1.65 scored higher. There was no difference between FIFA Elite (vs. National Elite), *t* = 0.84, *p* = 0.205. Similarly, on ATD, FIFA Super-Elite (vs. FIFA Elite), *t* = 2.351, *p* = 0.015, *d* = 1.61; and (vs. FIFA and National Elite), *t* = 2.687, *p* = 0.007, *d* = 1.42 scored higher. There was no difference between FIFA Elite (vs. National Elite), *t* = −0.07, *p* = 0.474, *d* = 0.03. There were no significant differences for CCS-M, *F*(2, 21) = 0.118, *p* = 0.890, η^2^ = 0.011 and MTQ10, *F* (2, 21) = 1.342, *p* = 0.283, η^2^ = 0.113 and planned comparisons found no differences between officiating groups (see Table 5).

**Table 5:** Planned comparisons examining conscientiousness, mental toughness, organizational culture, and athletic talent development scores relating to referee level.

Variable	Univariate ANOVA				Contrast								
					FSE vs. FE			FE vs. NE			FSE vs. F/NE		
	*F*	*df*	*sig*	η^2^	*t*	*sig*	*d*	*t*	*sig*	*d*	*t*	*sig*	*d*
CCS-M	0.118	2.21	0.890	0.01	0.48	0.318	0.35	−0.29	0.387	0.15	0.42	0.340	0.24
MTQ10	1.342	2.21	0.283	0.11	0.15	0.440	0.13	1.32	0.101	0.63	0.80	0.217	0.44
OCS	5.351	2.21	0.013	0.34	2.28	0.017	1.64	0.84	0.205	0.41	3.03	0.003	1.65
ATD	3.647	2.21	0.044	0.26	2.35	0.015	1.61	−0.07	0.474	0.03	2.69	0.007	1.42

FSE, FIFA Super Elite; FE, FIFA Elite; NE, National Elite; CCS-M, Concise Conscientiousness Measure; OCS, Organizational Culture Survey; ATD, Athletic Talent Development.

Having found an overall effect, further analysis examined subscales of the OCS and ATD in relation to FIFA Elite vs. FIFA Super-Elite using *t*-tests. Following Bonferroni correction, FIFA Super-Elite (vs. FIFA Elite) referees scored significantly higher on OCS subscales Information Flow and Involvement, and ATD subscales Long-Term Development Focus and Communication (Table 6).

**Table 6:** Differences between FIFA Super Elite and FIFA Elite referees on organizational culture and athletic talent development.

Variable	FIFA Super Elite		FIFA Elite		T-test Results			
	*M*	*SD*	*M*	*SD*	*t*	*df*	*sig*	*d*
Organizational culture								
Teamwork	23.80	5.40	18.71	3.82	1.92	10	0.042	1.23
Climate	21.20	2.77	15.71	4.35	2.46	10	0.017	1.58
Information flow	16.40	2.07	11.29	1.89	4.44	10	<0.001	2.85
Involvement	18.00	1.22	13.29	3.09	3.19	10	0.005	2.05
Supervision	28.00	4.95	23.86	5.43	1.35	10	0.103	0.87
Meetings	19.60	5.13	15.86	2.61	1.67	10	0.063	1.07
Athletic talent development								
Long-term development focus	21.60	2.51	17.14	2.91	2.76	10	0.010	1.77
Alignment of expectations	22.20	2.59	17.71	3.15	2.60	10	0.013	1.67
Comunication	21.00	3.46	15.14	3.29	2.97	10	0.007	1.91
Holistic quality preparation	17.60	7.92	15.29	5.02	0.62	10	0.274	0.40
Support network	20.40	2.70	17.00	3.46	1.82	10	0.049	1.17

## Discussion

The study investigated the influence of referee professional level on Athletic Talent Development (ATD) while controlling for the effects of Conscientiousness and Organizational Culture. Consistent with H1, we found a significant main effect. Furthermore, in support of H2, Conscientiousness and Organizational Culture were significant covariates, exerting medium and large effects on ATD, respectively.

Analysis revealed differences on ATD dimensions (i.e., Long-Term Development Focus, Alignment of Expectations, Communication, Holistic Quality Preparation, and Support Network). Post-hoc analyses showed that Level 4 referees, compared to Trainee–Level 5 officials, reported lower scores in Long-Term Development Focus and Holistic Quality Preparation. This suggests a shift in developmental experience occurs as referees transition from the initial, foundational stages (Trainee to Level 5) into officiating regional semi-professional leagues (Level 4).

In this context, Long-Term Development Focus refers to a commitment to continuous learning. Specifically, the environmental support for sustained growth and a clear career trajectory. Holistic Quality Preparation denotes a developmental context in which support extends beyond technical skill development to encompass integrated well-being. Thus, while trainees and new referees (Level 5) benefit from structured guidance and clear developmental goals, progress to Level 4, since it places a greater focus on performance, creates the perception of limited opportunities for learning, growth, and progression. Subsequently, the transition from Level 5 to Level 4 is associated with referees feeling less supported in terms of career planning and well-being, or a diminished propensity to engage with available support.

This finding identifies an officiating bottleneck (dip) at Level 4 in Long-Term Development Focus and Holistic Quality Preparation, where the immediate demands of regional semi-professional football overshadow long-term growth. This highlights a critical point beyond which many referees fail to advance. Indeed, within the sample, 74% of participants failed to move beyond Level 4. Thus, the performance-heavy environment and the associated perception of reduced developmental opportunities/support at Level 4 constitutes a barrier to referee retention and progression to higher levels.

This finding aligns with prior research (Giel and Breuer, 2020; Mojtahedi et al., 2024; Webb et al., 2020), which attributes the acute shortage of top referees to negative factors like insufficient administrative support. Consistent with this, Mola and Shaw (2024) emphasized the pivotal influence of management practices and organizational culture on talent development within sport. The perceived “Level 4 dip” in development focus and support within this study appears to be a specific manifestation of these systemic issues. To counter this, organizations must implement strategies that better integrate long-term development and well-being support into the Level 4 experience.

Exploration of differences between Elite and Super-Elite officials tested H3 and H4. While psychological attributes are essential for reaching FIFA level, consistent with H3, analysis found no significant differences in Conscientiousness or Mental Toughness between these groups. Instead, contextual factors, specifically the quality of the organizational environment and talent development system, differentiated them. In support of H4, FIFA Super-Elite (vs. FIFA Elite) referees expressed more positive perceptions of Organizational Culture and Talent Development. Further analysis revealed higher scores on Information Flow and Involvement facets of organizational culture, indicating enhanced sense of environment regarding greater transparency and shared decision-making. These referees also felt that their talent development environment was superior, specifically on the Communication, Long-Term Development Focus, and Alignment of Expectations subscales.

Higher scores on Communication suggest that top-tier officials benefit from a continuous, transparent dialog with their leaders, moving beyond basic messages to complex discussions. Elevated perceptions of Long-Term Development Focus and Alignment of Expectations signify that the personal ambitions of FIFA Super-Elite referees are more congruent with organizational strategies. Consequently, they view their careers as well-supported within the organizational framework.

Overall, findings suggest that the highest level of officiating is characterized by clear communication, strong support network, and a transparent pathway. This has important implications for how governing bodies manage their top officials, highlighting the critical role of the organizational framework in sustaining success at the pinnacle of the sport. Given the small number of officials at these top tiers, these findings require cautious interpretation, though the strong effect sizes found in the analysis support the need for further research.

Another difference was that Level 4 referees (vs. Level 3 through to FIFA International) scored significantly lower on Alignment of Expectations and Communication. Alignment of Expectations describes coherence between personal goals and organizational feedback and developmental pathways. Communication focuses on the clarity and effectiveness of dialog between officials and their coaches/mentors regarding development, training, and performance. This difference indicates that as referees reach elite levels, these factors become more refined. Hence, higher-tier officials experience more personalized goal setting and feedback, accompanied by direct and transparent discourse. In comparison, Level 4 officials perceive communications as less coherent, potentially contributing to a sense of uncertainty about their development trajectory. Consistent with the notion that Level 4 officials face distinct challenges, they scored lower (vs. trainee to Level 5 and Level 3 to FIFA International Level referees) on Support Network (i.e., perceived availability of developmental, emotional, and informational aid).

Collectively, these outcomes highlight meaningful differences in talent development perceptions associated with officiating experience. Such differences are important because they signal points in the pathway at which referees may benefit most from support and targeted intervention. In particular, provision that addresses challenges encountered at distinct stages of experience (e.g., managing pressure, handling conflict, adapting to rule changes) may facilitate continued progression and improve retention within the officiating system. Accordingly, understanding how perceptions of talent development vary across referee levels is valuable for governing bodies, as this insight can inform the design and delivery of support mechanisms (e.g., enhanced mentorship, clearer communication regarding promotion criteria) that promote sustained development and long-term involvement in officiating.

Beyond experience, analysis revealed that Conscientiousness, Mental Toughness, and Organizational Culture were inter-related and positively associated with perceptions of talent development. Organizational Culture demonstrated the strongest relationship with ATD, sharing approximately 25% variance (*r* = 0.50, *p* < 0.001). This finding aligns with prior research from Webb et al. (2023), which reported regional variations in mentoring and perceived support networks directly impacted referee promotion prospects and stress levels. Our results provide quantitative support for these qualitative findings, underscoring the vital role of the organizational environment in shaping perceptions of development. Specifically, a culture that encourages teamwork, effective information flow, high morale, and employee involvement is concomitant with an environment in which referees feel valued and supported, and able to develop.

Conscientiousness was also associated with ATD (sharing approximately 10% variance, *r* = 0.32, *p* < 0.001). This intimates that characteristics subsumed within the construct of conscientiousness (i.e., industriousness, control, perfectionism, and task planning) are associated with positive talent development pathway experiences. This finding supports previous research indicating conscientiousness is a principal feature of effective officiating (Dodt et al., 2022; Lampe et al., 2024; O’Brien et al., 2023). Our results extend this idea by suggesting conscientious referees not only excel in their on-field duties but by virtue of being initiative-taking also optimize engagement with developmental opportunities.

The relationship between Mental Toughness and ATD was weak (sharing approximately 2% variance, *r* = 0.12, *p* = 0.049), primarily correlating with Alignment of Expectations. While this weak association might seem surprising, it aligns with previous characterizations of mental toughness (Slack et al., 2013), which concern primarily the ability of referees to cope with immediate match-day pressures and adversity. This indicates that while essential for performance, mental toughness might be more of an individual characteristic that allows referees to cope with the demands of the environment, rather than a direct driver of how they perceive the quality of their development pathway. This implies a threshold effect, whereby a foundational level of mental toughness is a prerequisite for officiating at any level, but variations above this base do not significantly influence perceived talent development.

Exploration of differences between Elite and Super-Elite officials revealed nuanced findings. FIFA Super-Elite referees, compared to FIFA Elite and National Elite referees, scored higher on organizational culture and talent development perceptions. This indicates that the most accomplished officials view their organizational environments as more supportive, cohesive, and effective. Such perceptions may reflect access to enhanced resources (e.g., highly personalized coaching and mentoring, robust support pathways) designed to sustain world-class performance standards. In parallel, the organizational culture surrounding Super-Elite officials appears to prioritize continuous improvement, effective communication, and comprehensive support systems that enable consistent performance at the highest level. Overall, these distinct perceptions provide insight into the developmental pathways associated with achieving Super-Elite status and align with prior work differentiating Elite and Super-Elite performers (Güllich et al., 2019).

No differences in organizational culture perceptions were observed between FIFA Elite and National Elite referees, suggesting a potential threshold effect whereby top-tier officials experience a comparable level of organizational support. Likewise, perceptions of talent development appeared to differentiate only those who reach the very highest level, with Super-Elite referees reporting more favorable views than both Elite groups. Consistent with the organizational culture results, FIFA Elite and National Elite referees did not differ in their perceptions of talent development.

Across Super-Elite and Elite groups, there were no differences in conscientiousness or mental toughness. This suggests that while advancement to the highest levels of refereeing is associated with more positive perceptions of organizational support and talent development, core psychological attributes remain stable across these tiers. In other words, conscientiousness and mental toughness appear foundational for reaching elite status but do not differentiate between Elite and Super-Elite officials. These findings indicate that once a baseline of these psychological qualities is established, external systemic factors such as organizational culture and the quality of development pathways are likely to play a more influential role in differentiating those who reach the very highest level. Thus, the results highlight the importance of organizational support structures in nurturing and sustaining performance at the uppermost levels of officiating.

### Limitations

The researchers acknowledge study limitations. The use of a cross-sectional design, which gathered data at one point in time, prevented the establishment of causal relationships. Thus, while analysis revealed a strong association between Organizational Culture and ATD, we could not determine whether positive culture led to better ATD, or vice versa. To establish causal relationships, subsequent researchers should undertake longitudinal studies that collect multiple measurements over time, allowing for the exploration of changes and developments. This approach will provide a robust understanding of how study variables influence each other over an extended period.

Due to practical considerations (i.e., limited access to referees with time restrictions) the researchers used abridged scales within the study (e.g., MTQ10). While these possess attested psychometric properties, shortened versions assess limited construct breadth in comparison to full scale versions. Using abridged scales and/or using global rather than factor scores prevent researchers from examining within construct variations. These are necessary since they may reveal nuanced relationships, whereby overall differences are attributable to some rather than all factors. For instance, the MTQ10 is a unidimensional measure, which does not permit consideration of the relative contributions of control, commitment, challenge, and confidence. Similarly, by selecting only specific facets of conscientiousness (i.e., industriousness, control, perfectionism, and task planning), the study may have overlooked the potential influence of other facets (e.g., tidiness, procrastination refrainment, and caution). In addition, the combination of multiple potential comparisons and a constrained sample size of highly qualified referees raised statistical power considerations, which led the researchers to restrict analyses to global indices.

Building on this exploratory investigation, subsequent studies should accordingly target officials at higher levels and examine construct dimensions where appropriate. In the case of mental toughness and conscientiousness it appears that differences in referees overall as a function of level appear unlikely, however, consideration of facets of organization culture and talent development may further understanding of football officials’ perceptions and needs.

The use of self-report questionnaires to collect data was also problematic. Particularly, social desirability bias, where participants respond in ways they perceive as favorable or expected, rather than reflecting their actual perceptions or experiences, was a concern. While the researchers assured anonymity, within the Elite and Super-Elite groups some participants may have felt identifiable given the small number of highly qualified referees.

Common method bias, which refers to systematic error variance shared among measures due to their common method of collection (rather than true relationships between the constructs), was less of a concern in this study. While common method bias can distort observed relationships and produce misleading conclusions about the true strength or direction of an association, the researchers implemented methodological safeguards to mitigate its impact. Specifically, instructions to participants emphasized distinct section and construct differences, and the survey employed a range of response scales across the questionnaire. These techniques typically reduce the likelihood of common method bias.

Reliance on self-report affords only subjective perceptions of organizational culture and talent development. To determine how these translate into actual performance and/or career progression it is necessary to triangulate individual ratings with objective measures (e.g., match ratings, disciplinary records, fitness metrics, or actual promotion rates). For instance, while a referee might report a keen desire to utilize development opportunities they may not actively engage with training. Therefore, integrating self-report measures with objective measures in future research would provide a more robust evidence base.

A further consideration was the potential for researcher bias. One of the authors was a senior official, a proximity that informed the study by providing a deep understanding of the refereeing environment and facilitating access to high-level officials. However, this connection could unintentionally influence research design or interpretation, despite strict ethical protocols (e.g., ensuring anonymity and utilizing blind analysis) implemented to mitigate this risk.

Grouping Level 3 and higher officials into a single category, while necessary due to sample sizes at the higher levels, potentially obscured nuances in talent development experiences within the elite tiers. Though separate analyses of Super-Elite, FIFA Elite, and National Elite provided useful distinctions, a granular examination of each Level (e.g., Level 3, Level 2, National Elite, FIFA) in future studies, with larger numbers, could reveal additional specific developmental needs or challenges unique to each stage of progression.

The sample was predominantly male, and this imbalance limits the generalizability of the findings to female referees. The experiences, challenges, and talent development pathways for female officials may differ due to societal factors, physiological differences, and unique organizational support structures (Simpson et al., 2022; Theriault and Hancock, 2025; Tingle et al., 2021; Wicker et al., 2024). The current study cannot account for these variations, underscoring the need for future research to achieve gender-balanced or female samples to ensure broader applicability and provide insights relevant to all referees.

Other factors limiting generalizability included restricted demographic data and use of a convenience sample. Regarding demographic data, to ensure anonymity, the researchers collected only basic information (e.g., age, officiating level, and gender). Although this was crucial for protecting identity and encouraging participation it restricted consideration of demographic factors that influence talent development experiences and perceptions. In terms of sampling restrictions, findings are specific to football refereeing within the context of the study. While principles may be transferable to other refereeing contexts, direct generalization to specific bodies (i.e., local and national organizations) or sports requires caution since norms and structures vary considerably.

### Implications

This study highlights the multifaceted nature of referee progression, emphasizing that while individual psychological attributes are foundational, the perceived quality of the organizational environment is paramount. We found that progression to the highest levels of officiating is associated with increasingly positive perceptions of organizational support and talent development, while conscientiousness and mental toughness remain stable. Crucially, these data suggest a potential “bottleneck” in the developmental pathway, where mid-level officials perceive significantly less support compared to their lower and higher-level counterparts.

This underlines the need for officiating bodies to provide robust, consistent support to officials at all stages of their careers to improve recruitment, retention, and performance. Targeted interventions that address the specific challenges faced by referees at distinct experience levels (e.g., managing pressure, dealing with conflict, and adapting to rule changes) would facilitate continued progression and reduce attrition.

## Data Availability

The raw data supporting the conclusions of this article will be made available by the authors, without undue reservation.
